# Magnitude of anemia and associated factors among children aged 6–59 months at Debre Markos referral hospital, Northwest Ethiopia: a hospital-based cross-sectional study

**DOI:** 10.1186/s13052-021-01123-3

**Published:** 2021-08-13

**Authors:** Yoseph Merkeb Alamneh, Tadesse Yirga Akalu, Abtie Abebaw Shiferaw, Aytenew Atnaf

**Affiliations:** 1 Department of Biomedical Sciences, Debre Markos University, P.O. Box 269, Debre Markos, Ethiopia; 2 Department of Nursing, College of Health Science, Debre Markos University, Debre Markos Ethiopia, P.O. Box 269, Debre Markos, Ethiopia; 3 Department of Medical Laboratory Science, Debre Markos University, P.O. Box 269, Debre Markos, Ethiopia

**Keywords:** Anemia, Associated factors, Debre Markos, Northwest Ethiopia

## Abstract

**Background:**

Anemia, the most common micro-nutrient deficiency disorder, is the world’s second leading cause of morbidity and morbidity, affecting 24.8% of the population, of which 47.4% are under-five children. The prevalence of anemia ranges from 44 to 56% in Ethiopia. Although its magnitude has shown decreases across regions; it continues to be a significant public health problem, particularly in developing countries including Ethiopia. Despite this evidence, the magnitude and associated factors of anemia was not systematically explored and there is a limited information or limited evidences in the study area. Hence, the aim of this study was to assess the magnitude and associated factors of anemia among children aged 6–59 months attending at Debre Markos Referral Hospital, Northwest Ethiopia.

**Methods:**

A hospital-based cross-sectional study was conducted at Debre Markos referral hospital Northwest Ethiopia from September 30 to December 30, 2019. Data on socio-demographic and socio-economic factors, health and nutritional features of children and their mothers were obtained using pre-tested structured questionnaires in a face-to-face interview with child care providers. Blood samples and stool examination for intestinal parasites were performed. Hemoglobin level was analyzed using the HemoCue device (HemoCueHb 301). The collected data were coded, cleared and entered into Epi-Data version 3.1, and analyzed using Stata version 14 software. To identify candidates and predictor variables, bivariate and multivariate logistic regressions were applied respectively. The significance level was determined at a confidence interval of 95% at *p*-value < 0.05.

**Results:**

Of the total of 341 participants planned to be participated, about 310 mother-child pairs participated in the study, giving a response rate of 91%; and data were collected from children as well as their parents or guardians.

In this study, the magnitude of anemia was 11.9% (95% CI, 8.5, 16.2%). Poor dietary diversity (AOR = 2.3; 95% CI: 1.12, 5.14), food-insecure households (AOR = 3.24; 95% CI: 1.85, 4.52), complementary feeding initiation time (AOR = 3.20; 95% CI:1.23, 6.61), intestinal parasites infection (AOR = 3.20; 95% CI:1.23, 6.61) and family income (AOR = 2.87; 95% CI:1.57, 5.0) were found to be factors significantly associated with anemia.

**Conclusion:**

Overall, anemia is considered a public health problem among children aged 6–59 months attending at Debre Markos referral hospital, based on the cut-off point of the World Health Organization. Poor dietary diversity, complementary feeding initiation time, household food insecurity, intestinal parasite infection and family income were significantly associated with childhood anemia. Thus, it needs for proven interventions in public health such as food diversification, anti-helmintic drug provision and household food security. In addition, educating women about nutrition and diet diversification, as well as involving them in alternative sources of income-generating activity, can be vital in the study area**.**

## Introduction

Anemia can be defined as a reduction in the hemoglobin, hematocrit, or red cells number or their capacity to carry oxygen becomes insufficient to meet the physiological needs [1]. Anemia, the most common micro-nutrient deficiency disorder, can affect a person at all stages of life at any time, particularly among children aged 6–59 months and pregnant women, because of their higher iron requirements [2]. According to the World Health Organization (WHO) criteria, anemia is considered in terms of age and sex based on the average hemoglobin levels: Children from 6 months to 6 years old = 11 g / dl, Children aged 7 to 14 years = 12 g / dl, Adult men > 15 years of age = 13 g / dl, pregnant women = 11 g / dl, and non-pregnant women = 12 g / dl [3, 4]. In the population, anemia with a magnitude of 40%, 20–39.9%, 5–19.9% and < 5% is graded as severe, moderate, mild and no public health problem, respectively [5]. It is the world’s second leading cause of death and morbidity for the entire global burden of disease, affecting 1.62 billion people, accounting for 24.8% of the population, of whom 47.4% are children under 5 years of age [6, 7]. Anemia in children aged 6–59 months is a major health issue in nearly all developing world countries with an estimated magnitude of about 43%; with regional disparities of 62.3% in Africa, 53.8% in South East Asia and 21.9% in the Western Pacific region [4]. The magnitude of anemia in children ranges from 5% in North America to 22% in Europe and 30–63% in Asia, 12–58% in Africa [8, 9]. The magnitude of anemia among hospitalized children in various parts of Africa has been studied; 56.3% in Uganda; 83.2% in Southern Tanzania and 44–56% in Ethiopia [10–12]. In Ethiopia, anemia affects as many as six out of ten children under 5 years of age, but the proportion varies across the country due to food, income and culture disparities, for example, in the Somali region 83%, in Afar 75%, in Dire Dawa 72%, in the Amhara region 42% [13].

Anemia etiology is frequently multifactorial and interrelated in a complex manner, and anemia is correlated with socioeconomic, biological, environmental and nutritional factors such as stunting, low dietary diversity, food scarcity, timely initiation of supplementary feeding, deworming, wasting, educational status, parasite infestations, maternal weight and antenatal care visits [11, 14–18]. There are also numerous root causes of anemia, including blood loss, decreased RBC production, infectious diseases, maternal anemia during pregnancy, although 50% of its causes are iron deficiencies [19–22]. If left untreated, childhood anemia has severe consequences, including growth retardation, impaired motor and cognitive development, poor school performance, exposure to comorbid diseases, adult anemia predictors and increased morbidity and mortality [23, 24]. It also has adverse effects on economic development, physical, mental and social health [25].

Even its magnitude has shown decreases across regions; it continues to be a significant public health issue worldwide [26]. Although few local studies have shown magnitude of anemia, data on children are not available particularly in our study area. To minimize the anemia impacts, this section of the population needs to be stressed in order to provide an integrated package of interventions. Thus, evidence-based development of possible strategies and response to the problem will contribute significantly. In addition, it is a paramount importance to generate epidemiological data, identify risk factors and determine the magnitude of the anemia that regional and national public policies are being reformed; it is also important for program planners and policy makers to develop adaptable intervention and control strategies for the local context. Furthermore, these factors are more prevalent and with greater focus on the prevention aspects of anemia, concrete evidence is created in which evidence can encourage policymakers and program managers to implement effective intervention to monitor and mitigate the negative consequences of childhood anemia. However, as far as the authors are aware, there was no previous studies in the study area. This study was therefore aimed to explore the magnitude of anemia and identify associated factors among children aged 6–59 months at Debre Markos referral hospital, Northwest Ethiopia.

## Materials and methods

### Study design, period and area

A hospital-based cross-sectional study was conducted at Debre Markos referral hospital which is located at Debre Markos town, 299 km northwest of Addis Ababa, the capital city of Ethiopia. The study period was from September 30 to December 30, 2019. The town is situated at a latitude and longitude of 10° 21′N and 37°42′E, respectively, with an elevation of 2446 m above sea level [27]. According to the 2007 population and housing census of Ethiopia, the projected total population of the town was 132,363, of which 66,314 (50.5%) are female and 66,049 (49.5%) are male [28].

### Study population

All under-five children attending at Debre Markos referral hospital. This study included all children between 6 months and 59 months of age attending at Debre Markos Referral Hospital during the study period. However, children suffered from severe medical or surgical conditions, severe bleeding, iron and vitamin A supplementation over the past 3 months and blood transfused for less than 3 months were excluded from the study.

### Sample size determination and sampling technique

The sample size was determined using a single population ratio formula, and calculated using Open Epi-Data Version 3.1. The following statistical assumptions were considered for calculating our sample size: *p* = magnitude of anemia 28% taken from a study conducted in Ethiopia [29], Z α/2 = the corresponding Z score of 95% CI and d = Margin of error (5%).

$$ {\displaystyle \begin{array}{c}\frac{\mathrm{n}={\left(\mathrm{z}\upalpha /2\right)}^2\times \mathrm{p}\ \left(1-\mathrm{p}\right)}{{\mathrm{d}}^2}\\ {}\frac{\mathrm{n}={(1.96)}^2\times 0.28\left(1-0.28\right)=310}{(0.05)^2}\end{array}} $$

Finally, the final sample size of our analysis was estimated as 341 after adding 10% of none response rate. It was intended to include all 341 children aged 6–59 months who visited at Debre Markos Referral Hospital consecutively during the study period.

### Data collection tools and procedures

Data were collected via questionnaire administered by interviewees, prepared in English and translated into Amharic language. A pretested structured questionnaire was used through a face-to - face interview to gather socio-demographic and economic data from mothers. The tools for data collection were developed through review of guidelines and related literatures [18, 30]. The design of the questionnaire included the following four sections. The first section dealt with the socio-demographic characteristics. The second part dealt with household food service status, assessed using the standardized questionnaire developed by Food and Nutritional Technical Assistance [31]. Part three was designed to identify child feeding practices, the modified version of the Helen Keller International Food Frequency Questionnaire and the 24-h dietary recall were used to identify dietary patterns and dietary diversity scores, respectively, and the fourth part assessed children’s morbidity [32, 33].

### Measurement of variables

#### Hemoglobin determination and anemia diagnosis

Concentration of hemoglobin was used to determine the anemia status by taking capillary blood samples. Hemoglobin level was analyzed using the HemoCue device (HemoCueHb 301) and altitude values have been adjusted using WHO guidelines. Anemia has been characterized as having a hemoglobin level below 11 g / dl. It has been further graded into mild, moderate, and severe anemia. Mild anemia was 10–11 g / dl for children with hemoglobin levels while mild anemia was 7–10 g / dl for children with Hb levels and severe anemia was below 7 g / dl for children with hemoglobin levels. The hemoglobin concentration below 11.0 g / dl was considered anemic whereas the hemoglobin concentration 11.0 g / dl or higher was considered normal. Anemia severity was classified according to the WHO guideline as follows: children were categorized as mild, moderate and severe anemic if their blood hemoglobin concentrations are between 10.0–10.9 g / dl, 7.0–9.9 g / dl and < 7.0 g / dl, respectively [18].

#### Laboratory analysis

For the analysis of hematological parameters, about 2 ml of venous blood was collected from each subject; hemoglobin (Hgb) was determined using the HemoCue device (HemoCueHb 301). To examine hemiparasites, thick and thin films of blood were prepared and stained with Giemsa stain. Stool sample was collected and examined for intestinal parasite infection using both direct wet mounting techniques and formol ether concentration.

#### Outcome variable

Hemoglobin level below 11 mg / dl was taken as dependent variables.

#### Independent variables

Socio-demographic and socioeconomic characteristics: sex of child, age of child (months), age of mother (years), residence, occupation of the mother, number of under-five children, maternal educational status, annually family income; health and nutritional characteristics of children aged 6–59 months and their mothers: initiation of complementary feeding, complementary feeding frequency/day, intestinal parasites,, dietary diversity score, vaccination status, antenatal care follow-up, place of delivery, maternal anemia, household food insecurity, mothers’ meal frequency / day were independent variables.

### Data quality assurance and control

The questionnaire was prepared in English, translated into the local (Amharic) language and then translated back into English to check consistency. After training was given on the study objective, consenting, data collection techniques, data were collected by trained data collectors. Investigators closely monitored the data collection process. All measurements were taken by following the recommendation of the manufacturers. All percentage of technical error measurement (%TEM) values for either inter- or intra-observer reliability of anthropometric data measurement was classified as ‘acceptable’ (< 2%), and coefficients of reliability greater than 99%.

### Data management, analysis and interpretation

Data were entered into the Epi-Data version 3.1 software and analyzed with Stata version 14 software. Data cleaning was carried out to check the frequency, accuracy, consistency and missed values; any identified errors were corrected. Proportions, means and standard deviations (SD) were used to describe independent variables and anemia in the study population. In order to identify candidate variables (*p*-value < 0.25) for multivariate regression, bivariate logistic regression was done using independent variables. In the bivariate analysis, variables with a *p*-value of < 0.25 were fitted into the multivariate analysis of binary logistic regression. Finally, predictors of anaemia were determined among the selected candidate variables using multivariate logistic regression model. Interaction, mediation, and multi co-linearity between independent variables were checked on the basis of assumptions such as tolerance, variance inflation factors (VIF), interaction correlation coefficient and others to ensure the fitness of the logistic regression model. Multicollinearity was assessed by examining Variance Inflation Factor (VIF) and others, which assesses how much the variance of an estimated regression coefficient increases if predictors are correlated, a value of VIF were in acceptable range (less 5), indicates that there is no correlation between this independent variable. The final model was tested for its fitness by Hosmer and Lemeshow *p*-value and *p*-value was 0.73 considered good fitness of the model [34]. To show the strength of the association, both the crude odds ratio (COR) and the adjusted odds ratio (AOR) with the corresponding 95% confidence interval (CI) were calculated. The statistical significance was determined in the multivariate analysis at a confidence interval of 95% at *p*-value < 0.05.

### Ethical approval and consent

The actual gathering of data was started after the study was approved by Debre Markos University. Local administrative bodies were also communicated about the study, and permission was obtained prior to the study. Finally, informed written consent obtained from the mothers / caregivers.

## Results

### Socio-demographic and socioeconomic characteristics

In this study, a total of 310 mother-child pairs were participated, giving a response rate of 91%. Majority of the respondents were male 162(52.3%). The age range of children was 6 to 59 months and the mean ages were 28.7 ± 4.302 months. Most of study participants (64%) have greater than two children and about 53.3% had greater than annual income of 10,000 ETB (Table 1).

**Table 1:** Socio demographic characteristics of children age 6–59 months at Debre Markos Referral Hospital, Northwest Ethiopia, 2019

Variables (***n*** = 310)	Categories	n (%)
Sex of child	Male	162 (52.3)
	Female	148 (47.7)
Age of child (months)	6–11	62 (20.0)
	12–23	83 (26.8)
	24–35	67 (21.6)
	36–47	47(15.2)
	48–59	51(16.5)
Age of mother (years)	15–29	182 (58.7)
	30–49	128 (41.3)
Residence	Urban	209 (67.4)
	Rural	101(33.6)
Occupation of the mother	House wife	108 (34.8)
	Self employed	33(10.7)
	Employed	93 (30.0)
	Farmer	76 (24.5)
Number of under-five children	One child	113 (36.5)
	Two & above children	197 (64.5)
Maternal educational status	Unable to read & write	70 (22.6)
	Primary school	57 (18.4)
	Secondary school	73 (23.5)
	College/ University	110 (35.5)
Annually family income (ETB)	< 10,000	145 (46.7)
	> 10,000	165 (53.3)

### Health and nutrition characteristics

Among the study participants, about two hundred and nineteen children (70.6%) started complementary feeding at the 6th month and above. Moreover, the children had a mean dietary diversity score of 3.4 with a standard deviation of 1.5, and only 124 (40%) consumed more than half of the recommended seven food groups. The magnitude of maternal anemia was 30.3%, of which 233 (75.2%) consumed less than four meals a day. Seventy-eight (25.1%) mothers fully attended the four ANC visits recommended by the WHO, while only 48 (15.5%) did not have an ANC visit. Based on the Measurement of Household Food Insecurity Access Scale (HFIAS), 140 (45.2%) of children were born and live-in food-secure households, while the rest were born and live-in food-insecure households (Table 2).

**Table 2:** Health and nutritional characteristics of children aged 6–59 months and their mothers at Debre Markos Referral Hospital, Northwest Ethiopia, 2019

Variables (***n*** = 310)	Categories	n (%)
Initiation of complementary feeding	< 6 months	91 (29.4)
	> 6 months	219 (70.6)
Complementary feeding frequency/day	≤ 3	202 (65.2)
	> 3	108 (34.8)
Intestinal parasites	Yes	214 (69.0)
	No	96 (31.0)
Dietary diversity score	Below 4	186 (60)
	4 and above	124 (40)
Fully vaccinated	Yes	273 (88.0)
	No	37 (12.0)
Antenatal care follow-up	No visit	48 (15.5)
	1–3 visit	184 (59.4)
	4 and above	78 (25.1)
Place of delivery	Home	130 (41.9)
	Health facility	180 (58.1)
Maternal anemia	Yes	94 (30.3)
	No	216 (69.7)
Household food insecurity	Food secure	140 (45.2)
	Mildly food insecure	50 (16.1)
	Moderately food insecure	78 (25.2)
	Severely food insecure	42 (13.5)
Mothers’ meal frequency / day	≤ 3	233 (75.2)
	> 3	77 (24.8)

### Magnitude and severity of anemia

The overall magnitude of anemia among children aged 6–59 months was, 11.9% (95% CI: 8.5–16.2%) with hemoglobin levels below 11 g/dl. Among anemic cases, the majority were mild (10–10.9 g / dl) and moderate (7–9.9 g / dl) anemics with 22 (7.1%) and 11 (3.5%) respectively; while only 4 (1.3%) children were seriously anemic with hemoglobin levels below 7 g / dl.

### Factors associated with anemia

Based on bivariate logistic regression, anemia among children aged 6–59 months was associated with age of child, age of mother, annual family income, complementary feeding initiation period, intestinal parasites, dietary diversity score and food insecurity (*p* < 0.05). However, in the multivariable logistic regression analysis, children’s age, mothers’ age was not associated with childhood anemia (*p* > 0.05); whereas in the multivariable logistic regression analysis, the initiation time of complementary feeding, intestinal parasites, annual family income, dietary diversity score and household food insecurity were identified as predictors of child anemia (*p* < 0.05). Depending on the multivariable logistic regression logistic regression, the nutritional aspects of childhood anemia have also been found to be predictive factors. Children consuming poor dietary diversity (DDS < 4) were therefore found to be more likely to be anemic than their counterparts (AOR = 2.3; 95% CI: 1.12, 5.14). In addition, children from food deprived households were three times more likely to be anemic than those from food-secure households (AOR = 3.24; 95% CI: 1.85, 4.52). The odds of developing anemia among children aged 6–59 months with annual family income of ≤10,000 ETB were nearly three times higher compared with annual family income of > 10,000 ETB (AOR = 2.87; 95% CI:1.57, 5.0). Similarly, starting of the complimentary feeding before 6 months, the chances of anemia were three times higher than those of children who started supplemental feeding after 6 months. In addition, the chances of developing anemia were three times higher among individuals with intestinal parasites (AOR = 3.20; 95% CI:1.23, 6.61). (Table 3).

**Table 3:** Bivariate and Multivariable analysis of factors associated with anemia among children aged 6–59 months at Debre Markos referral hospital, Northwest, Ethiopia, 2019

Variables	Categories	Anemia status		COR (95% CI)	AOR (95% CI)	*P* value for AOR
		Anemic n (%)	Non anemic n (%)			
Sex of child	Female	20(13.5%)	128(86.5%)	1.33(0.67–2.65)	–	–
	Male	17(13.5%)	145(86.5%)	1		
Childs’ age in months	6–11	15(40.5%)	47(59.5%)	4.68(1.27–17.3) *	3.62(0.83–6.5)	0.849
	12–23	9(24.5%)	74(76.5%)	1.78(0.46–6.94)	2.34(0.40–13.6)	0.343
	24–35	5(13.5%)	62(86.5%)	1.18(0.27–5.21)	0.83(0.12–5.56)	0.778
	36–47	3(8.1%)	44(91.9%)	1.59(0.36–7.07)	0.76(0.12–4.88)	0.171
	48–59	5(13.5%)	62(86.5%)	1	1	
Mothers’ age in years	15–29	21(11.5%)	161(89.5%)	1	1	
	30–49	16(12%)	128(89.1%)	4.13(2.6–18.3) *	2.54 (0.35–5.34)	0.471
Residence	Urban	17(8.1%)	192(91.9%)	1	1	
	Rural	20 (19.8%)	81(80.2%)	2.79(1.39–5.60)	2.52(0.67–9.47)	0.114
Mothers’ occupation	employed	9(7.1%)	117(92.9%)	1	1	
	House wife	10 (9.3%)	98(90.7%)	0.25(0.11–0.59)	4.50(0.69–29.1)	0.818
	Farmer	18(23.7%)	58(76.3%)	0.33(0.14–0.760)	1.17(0.30–4.61)	0.707
mothers’ educational Level	Unable to read	22(31.4%)	48(68.6%)	0.162(0.7–0.38)	0.10(0.03–0.38)	0.234
	Primary and secondary school	9(6.9%)	121(93.1%)	0.13(0.05–0.33)	0.07(0.01–0.47)	0.206
	College & above	6(5.5%)	104(94.5%)	1	1	
Annual family income	≤10,000 ETB	30(20.7%)	115(79.3%)	5.89(2.50–13.87) *	2.87(1.57–5.0) **	**0.004**
	> 10,000 ETB	7(4.2%)	158(95.8%)	1	1	
Complimentary food starting	< 6 months	20(21.9%)	71(79.1%)	3.35(1.66–6.75) *	3.38(1.32–8.64) **	**0.016**
	> 6 months	17(7.8%)	202(92.2%)	1	1	
Intestinal parasites	Yes	198(92.5%)	16(7.5%)	3.45(0.72–6.99) *	3.20 (1.23–6.61) **	**0.024**
	No	21(21.8%)	75(78.2%)	1	1	0.445
Child history of anemia	Yes	2(33.3%)	4(66.6%)	1	1	
	No	35(11.5%)	269(88.5%)	0.26(0.05–1.47)	2.82(0.20–40.4)	0.317
Place of delivery	Home	8(6.2%)	122(93.8%)	1.71(0.69–4.22)	2.54(0.56–5.81)	0.158
	Health facility	29(10.1%)	151(89.9%)	1	1	
Hospital stay of child	1 day	32(11.7%)	241(88.3%)	1.18(0.43_3.24)	–	–
	> 1 day	5(13.5%)	32(86.5%)	1		
Number of Children	One child	10(8.8%)	103(91.2%)	1	1	
	2 &abo child	27(13.7%)	170(86.4%)	0.61(0.28–1.32)	1.416(0.44–4.55)	0.559
Dietary diversity score	Below 4	99	87	6.6(3.76–11.59) *	2.3(1.12–5.14) **	**≤ 0.001**
	4 and above	20	104	1	1	
Household food insecurity	Yes	55	85	1	1	
	No	87	163	1.21 (0.8–1.86)	3.24 (1.85–4.52) **	**≤ 0.001**

*significant variables of bivariate analysis

**significant variables of multivariate analysis

## Discussion

The purpose of this study was to determine the magnitude and associated factors of anemia in children aged 6–59 months who visited Debre Markos Referral Hospital in Northwest Ethiopia. Despite the progress of nutritional interventions, anemia remains one of the major health problems in developing countries resulting in serious health outcomes, particularly among children aged 6–59 months and pregnant women [2]. It is a moderate to severe public health issue that has been significantly associated with negative effects on the overall health of children, social and economic development [35, 36]. According to the report by the WHO and the United Nations on progress towards the Millennium Development Goals (MDGs), although significant progress has been made towards achieving MDG4 to reduce the number of under-five mortality rates worldwide, the rate of decline remains insufficient to meet the stated goal in developing countries,including Ethiopia [37, 38]. This raises concerns about the future and effectiveness of interventions that reduce anemia, because the magnitude of anemia is a powerful indicator for assessing the impact and effectiveness of interventions [2].

In this study, the overall magnitude of anemia among children aged 6–59 months was found to be 11.9% (95% CI: 8.5–16.2%). It is a public health problem that should be addressed using appropriate preventive strategies as anemia contributes to childhood morbidity and mortality [39]. According to the WHO, if the magnitude is greater than 5%, anemia is considered a public health problem, however, the magnitude of the problem is defined as mild, moderate and severe when the magnitude is 5–19.9%, 20–39, 9% and ≥ 40% respectively [40]. Consequently, there is a mild public health problem in the study area that should be addressed using appropriate preventive methods as anemia leads to child morbidity and mortality [39]. This finding is comparatively close to other local studies carried out at Mekelle (11%) [30, 41], but higher than the study done at Addis Ababa (5.83%) [42]. Also this study was comparatively close to other local studies carried out in Lithuania (10.1%) [43], Serbia (10.8%) [44], Mexico (12%) [45], and Brazil (9.3%) [41]. This could be explained by the high magnitude of chronic early childhood undernutrition in study area based on the 2016 DHS reports [46]. Furthermore, the possible explanations for variations in the magnitude of anemia between the present study and the above studies may be related to the seasonal and geographical variation of risk factors and disparities in the socioeconomic status of the populations.

The current study findings were lower than the studies done at Kersa (27.1%) [47], Filtu (23.66%) [48], Libo-Kemkem and Fogera districts (30.9%) [49], Kenya (28.8–35.3%) [50], West Africa (23.8%) [51], China 34% [52]. The potential reason for this study’s low magnitude of anemia may be attributed to variations in the research timeframe and the impact of the biannual deworming system which is a major public health initiative recently initiated by government to minimize intestinal infection by offering antihelmintics.

In this research, household income has also been associated with anemia among children. Children living in households with lower monthly incomes were more likely to suffer from anemia than those with higher incomes. This is supported by similar findings in a study carried out in Brazil and northern Ethiopia [53, 54]. This is because children from poor families are less likely to get iron-rich foods such as animal products and less likely to be able to afford health care during illness due to poverty. It is, therefore, necessary to engage women in income-generating activities so that their children have better health care and supplementary food.

In addition, children eating low dietary diversity were found to be more likely to be anemic than their counterparts according to this report. Children who were given less than four food categories per day were 1.71 times more likely than their peers to develop anemia, and children who were food insecure were 2.87 times more likely than their counterparts to develop anemia [55, 56]. This might be attributed to the absence of nutritious meals with high protein quality, enough micronutrient content and bioavailability, macro-minerals, iron, and essential fatty acids in children from food insecure homes, all of which raise the risk of childhood anemia [57]. This might be attributed to the seasonal shortage of citric fruits, which aid iron absorption, as well as social and economic constraints to obtaining animal-based meals like meat. As a result, it is essential to educate the community about the need of giving iron-rich foods to all children.

Similarly, beginning of the complimentary feeding before 6 months, the chances of anemia were three times higher than those of children who started supplemental feeding after 6 months. This study is consistent with a study conducted in northern Ethiopia, Lebanon, Brazil and China [14, 58–60], early solid or liquid meal introduction is linked to childhood anemia. Infections and mal-absorption are more likely in newborns who are exposed before the age of 6 months. This might be due to a lack of awareness about the necessity of exclusive breast feeding for infants; as a result, they introduce cow milk at least 6 months earlier. Consequently, the early initiation of supplementary feeding like cow’s milk before the age of 6 months does not substitute iron-rich diets, which may lead to iron deficiency anemia [61].

Once more, the magnitude of anemia was substantially high in children with intestinal parasite infection, which is confirmed by research from Tanzania and Nigeria [62, 63]. This may be because the majority of intestinal parasites, particularly hookworm and hemoparasites like malaria, contribute to blood loss and/or red cell destruction and thus contribute to anemia**.** Deworming is thus an essential intervention in children because intestinal parasites, particularly hookworm infection, lead to blood loss in the intestines, which in turn contributes to anemia. This study has some limitations: The first limitation of this study is that subclinical infections other than intestinal parasites and malaria have not been evaluated, thus limiting the study’s generalizability for possible risk factors**.** This research is quantitative; if qualitative approach was also used it would be stronger to examine the extra determinants of anemia. This research was often revealing of maternal bias as they recalled their previous past. Also, anthropometry-related measurement errors may have been introduced. Using small samples, it’s difficult to generalize to society at large. Therefore, larger studies need to demonstrate true associations in the population. Despite these limitations, it is the first hospital-based cross-sectional study that has attempted to show the magnitude and associated factors of childhood anemia severity in the study area, which contributes greatly to improving the health of children aged 6–59 months.

## Conclusion

Overall, anemia is considered a public health problem among children aged 6–59 months attending at Debre Markos referral hospital, based on the cut-off point of the World Health Organization. Poor dietary diversity, complementary feeding initiation time, household food insecurity, intestinal parasite infection and family income were significantly associated with childhood anemia. Thus, this needs for proven interventions in public health such as food diversification, anti-helmintic drug provision and household food security. In addition, educating women about nutrition and diet diversification, as well as involving them in alternative sources of income-generating activity, can be vital in the study area. On top of that, more extensive longitudinal studies using a larger sample size, including analysis of all indices of red blood cells, red cell morphology, serum micronutrient levels and subclinical infections, should be conducted to establish a causal-effect relationship between anemia and its contributing factors.

## Data Availability

The datasets used and/or analyzed during the current study are available from the corresponding author on reasonable request.

## Abbreviations

AOR: Adjusted Odds Ratio
ANC: Antenatal care
BAZ: Body Mass Index–for-Age Z-scores
CI: Confidence Interval
COR: Crude Odds Ratio
DDS: Dietary Diversity Score
ETB: Ethiopian Birr HAZ Height-for-Age Z-scores
Hb: Hemoglobin
HFSS: Household Food Security Status
MDG: Millennium Development Goals
RBC: Red Blood Cell
SD: Standard Deviation
WHO: World Health Organization.
